# Supplementary material for: Açai (Euterpe oleracea Mart.) Seed Extract Induces ROS Production and Cell Death in MCF-7 Breast Cancer Cell Line
Source: Molecules. 2021 Jun 10;26(12):3546. doi: 10.3390/molecules26123546 (PMC8230419; doi:10.3390/molecules26123546)
Supplement: Supplementary file 1 [file molecules-26-03546-s001.zip › molecules-1237329-supplementary.pdf]

Supplementary Data

Chemical compounds identified by GNPS hits

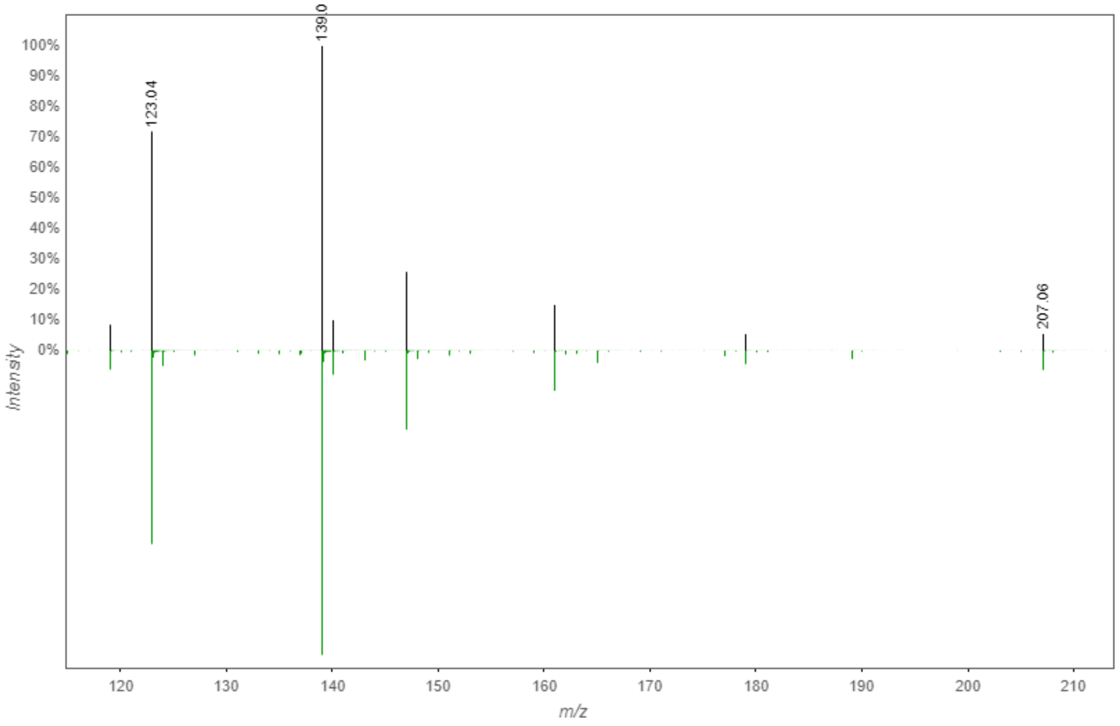

Fig S1. MS/MS match between GNPS database (green) and *Euterpe oleracea* Mart extract (black) for **1**.

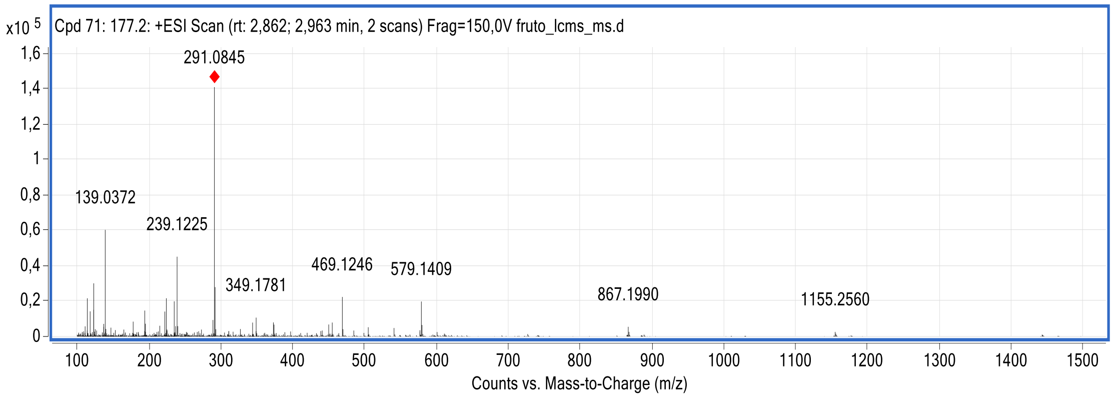

Fig S2. Mass Spectrum of $[M+H]^+$ of **1**.

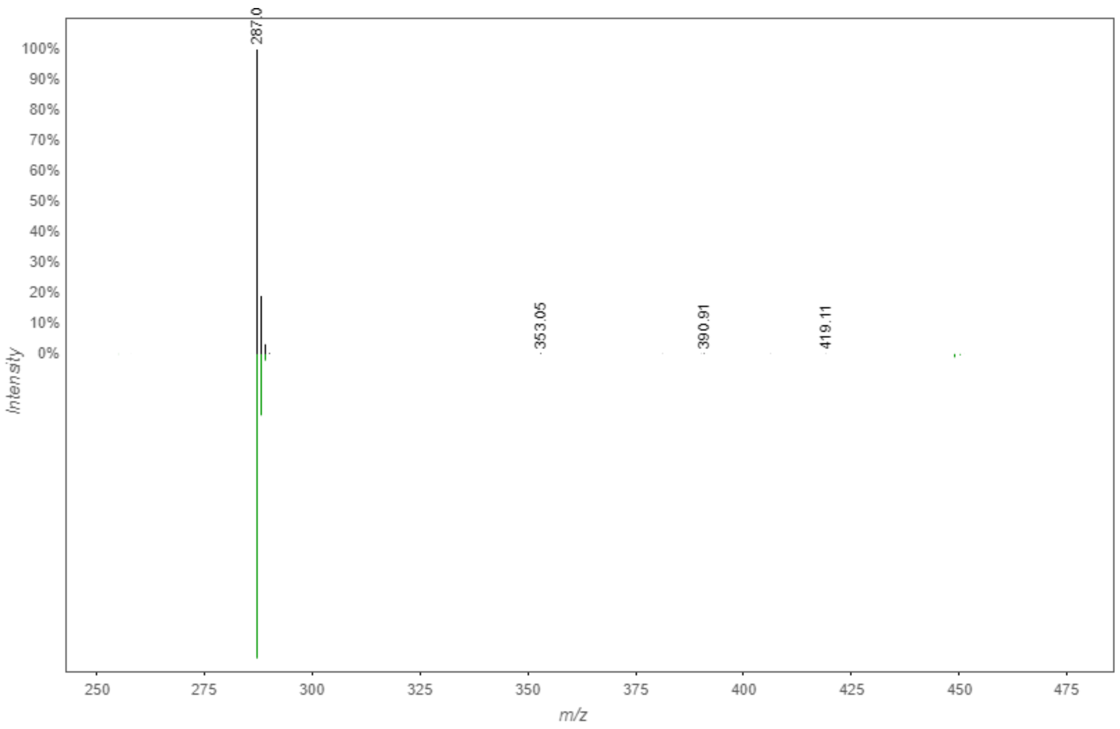

Fig S3. MS/MS match between GNPS database (green) and *Euterpe oleracea* Mart extract (black) for **2**.

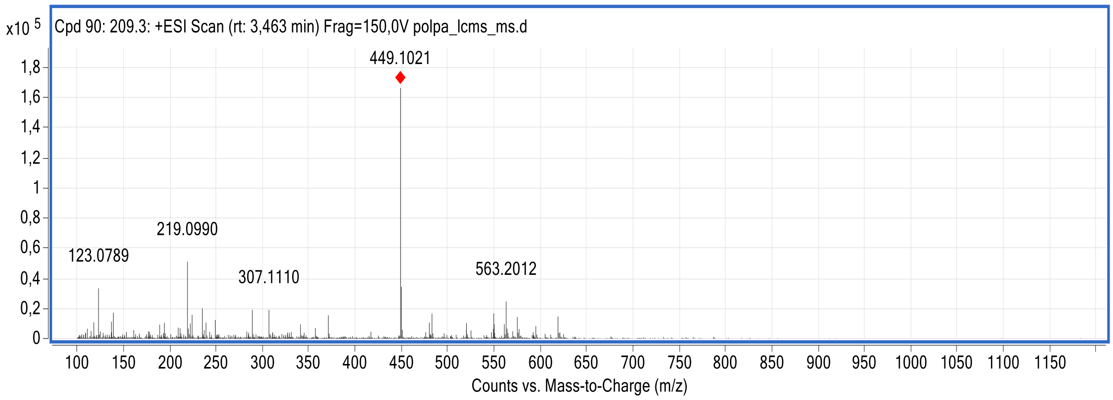

Fig. S4. Mass Spectrum of $[M+H]^+$ of **2**

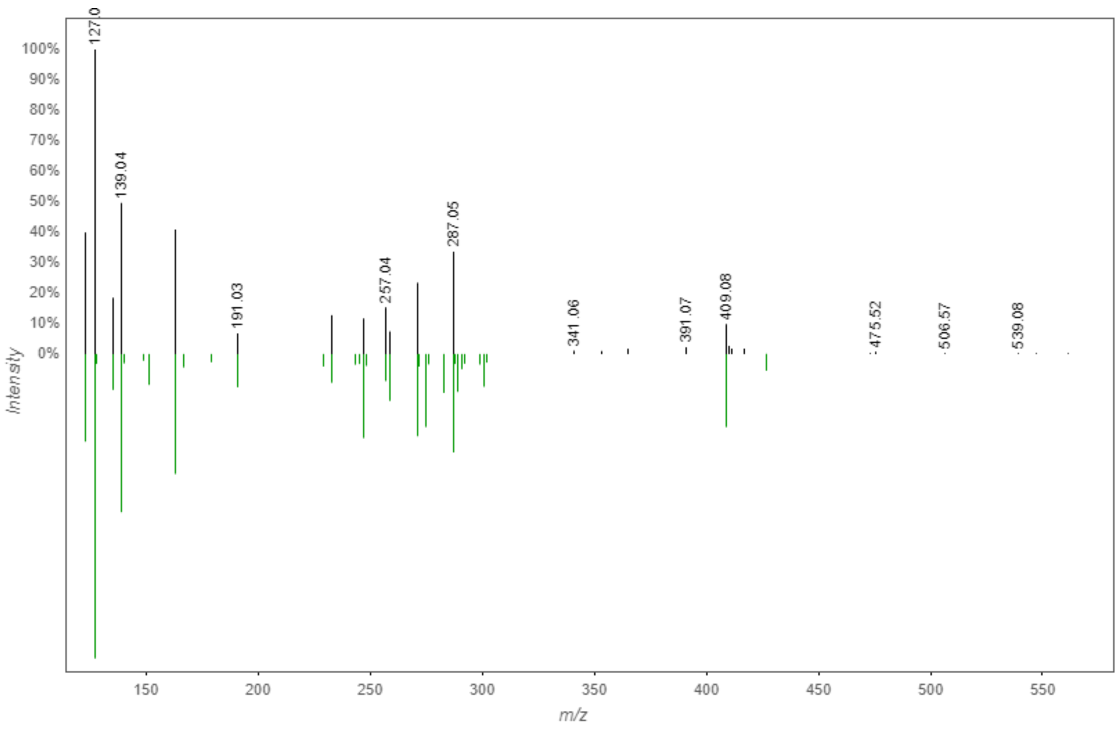

Fig S5. MS/MS match between GNPS database (green) and *Euterpe oleracea* Mart extract (black) for **3**.

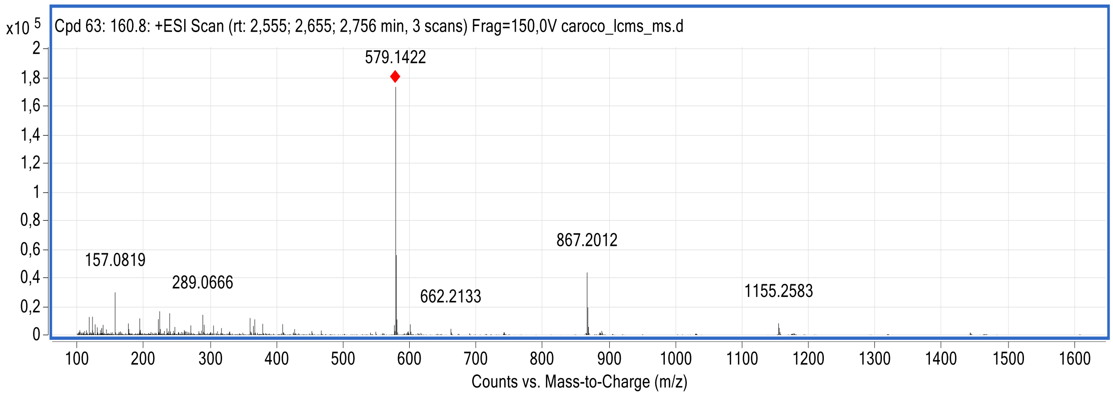

Fig. S6. Mass Spectrum of $[M+H]^+$ of **3**

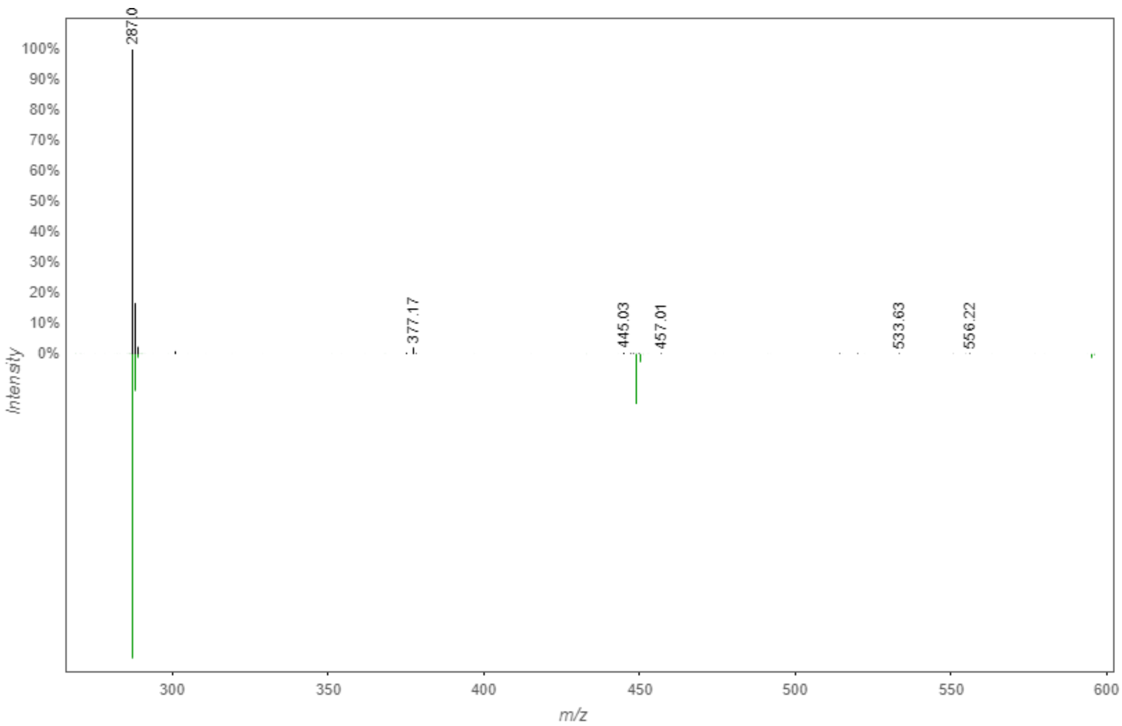

Fig S7. MS/MS match between GNPS database (green) and *Euterpe oleracea* Mart extract (black) for **4**.

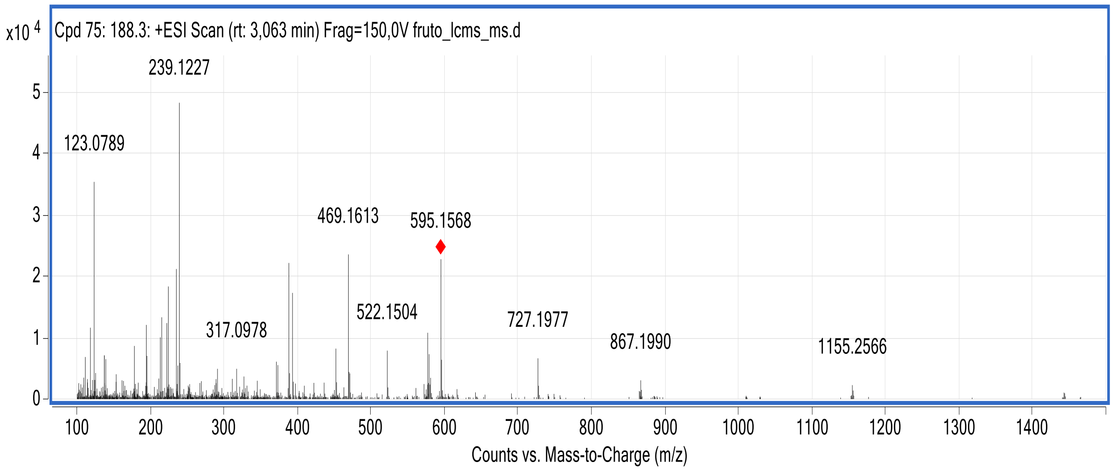

Fig. S8. Mass Spectrum of $[M+H]^+$ of **4**

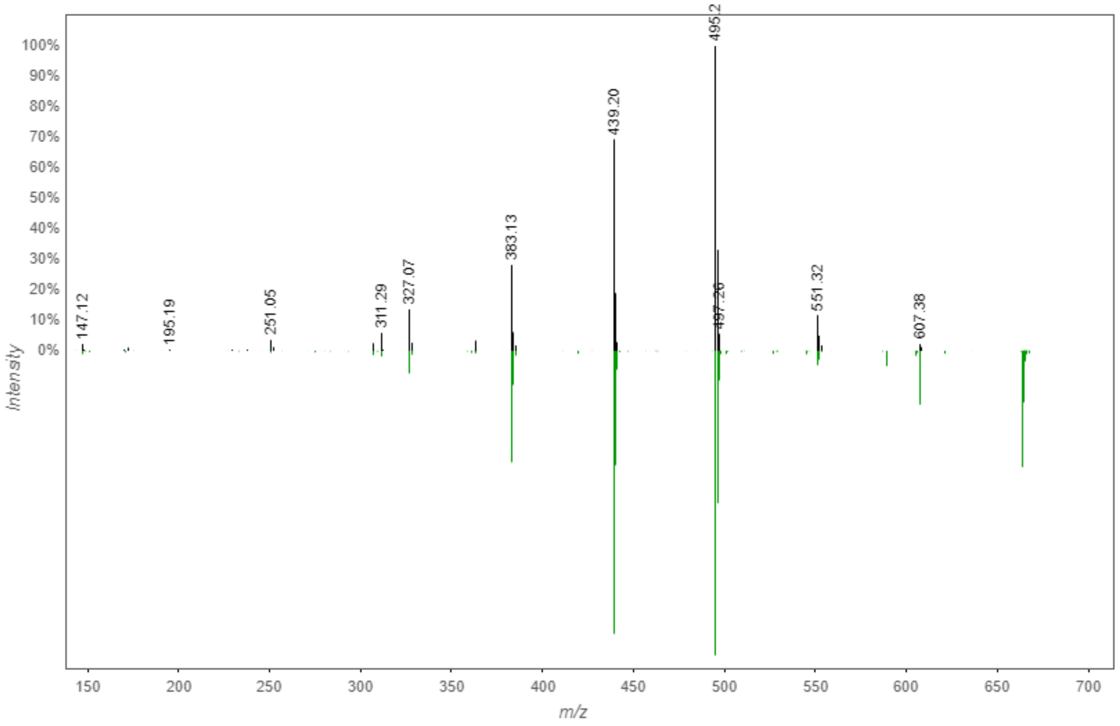

Fig S9. MS/MS match between GNPS database (green) and *Euterpe oleracea* Mart extract (black) for **5**.

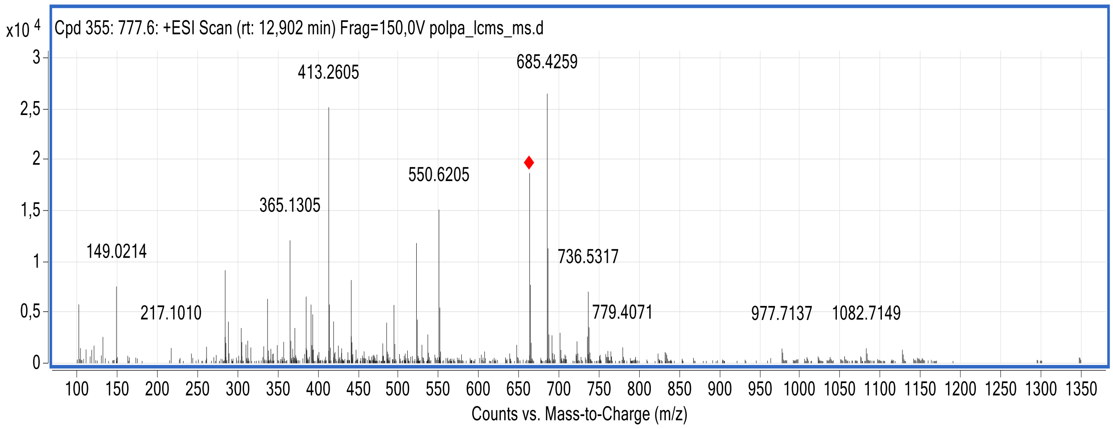

Fig. S10. Mass Spectrum of $[M+H]^+$ of **5**

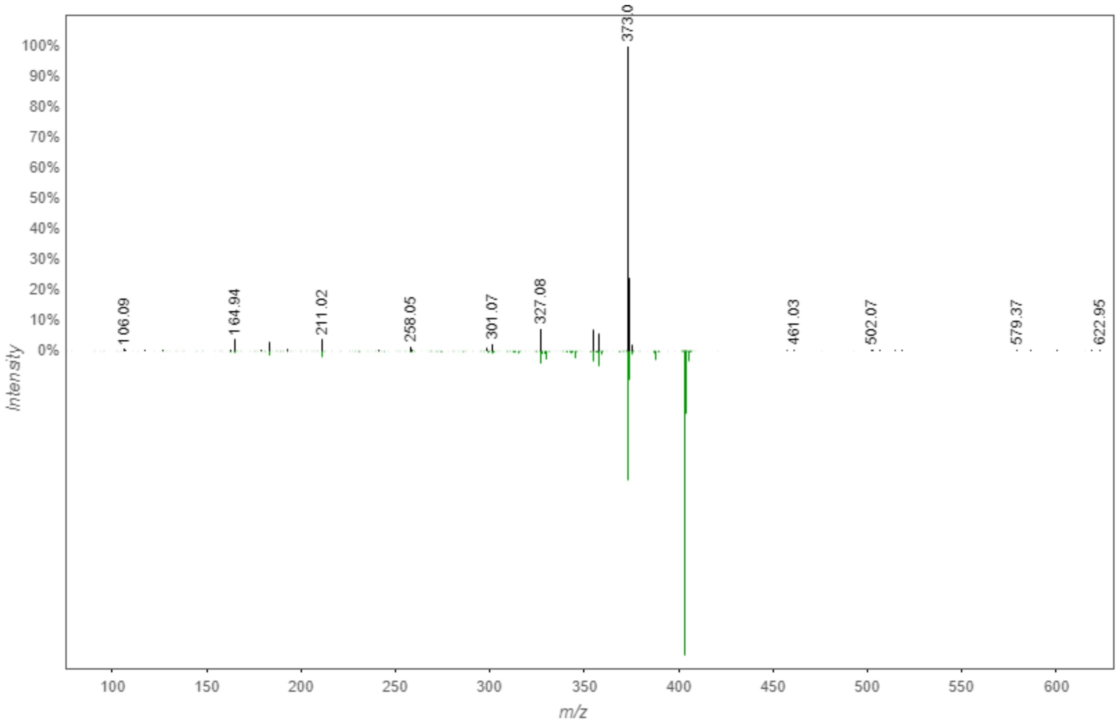

Fig S11. MS/MS match between GNPS database (green) and *Euterpe oleracea* Mart extract (black) for **6**.

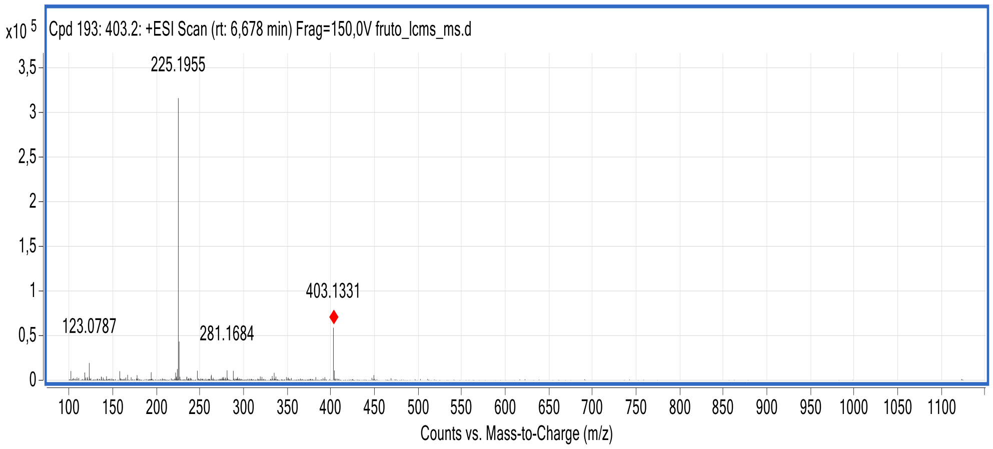

Fig. S12. Mass Spectrum of $[M+H]^+$ of **6**

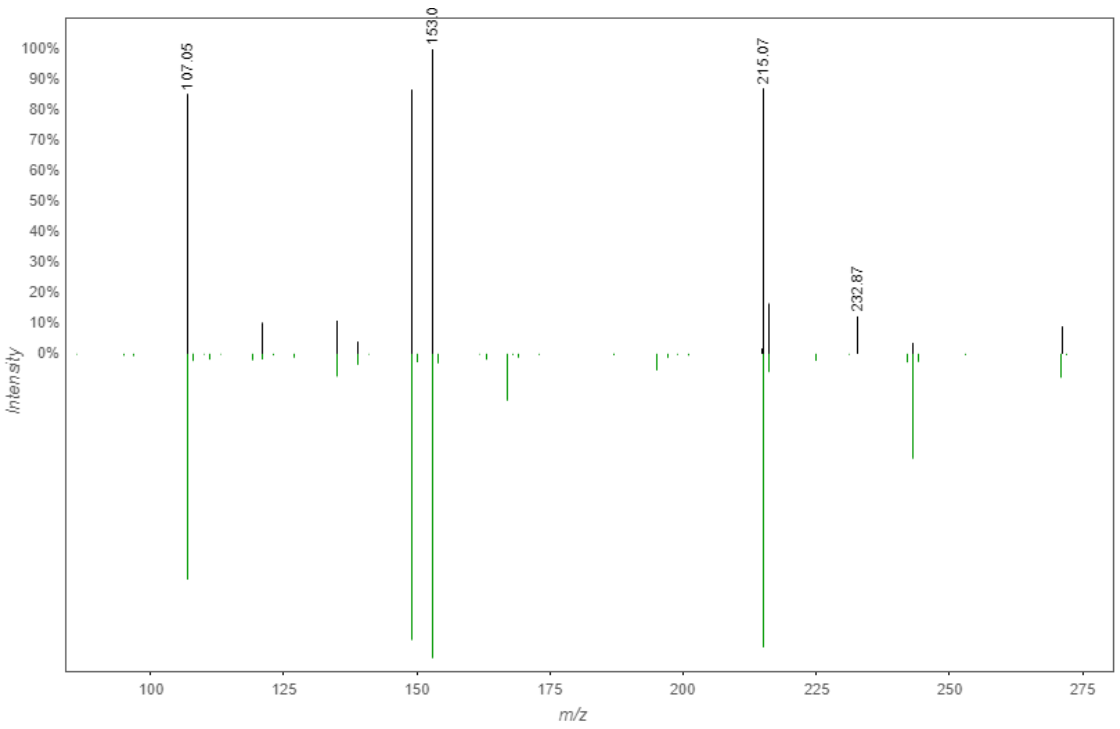

Fig S13. MS/MS match between GNPS database (green) and *Euterpe oleracea* Mart extract (black) for **7**.

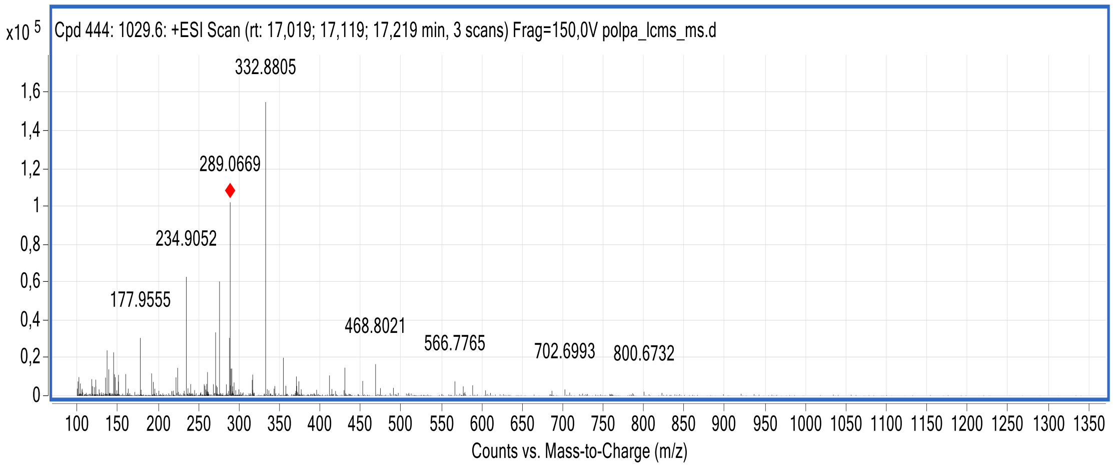

Fig. S14. Mass Spectrum of $[M+H]^+$ of **7**

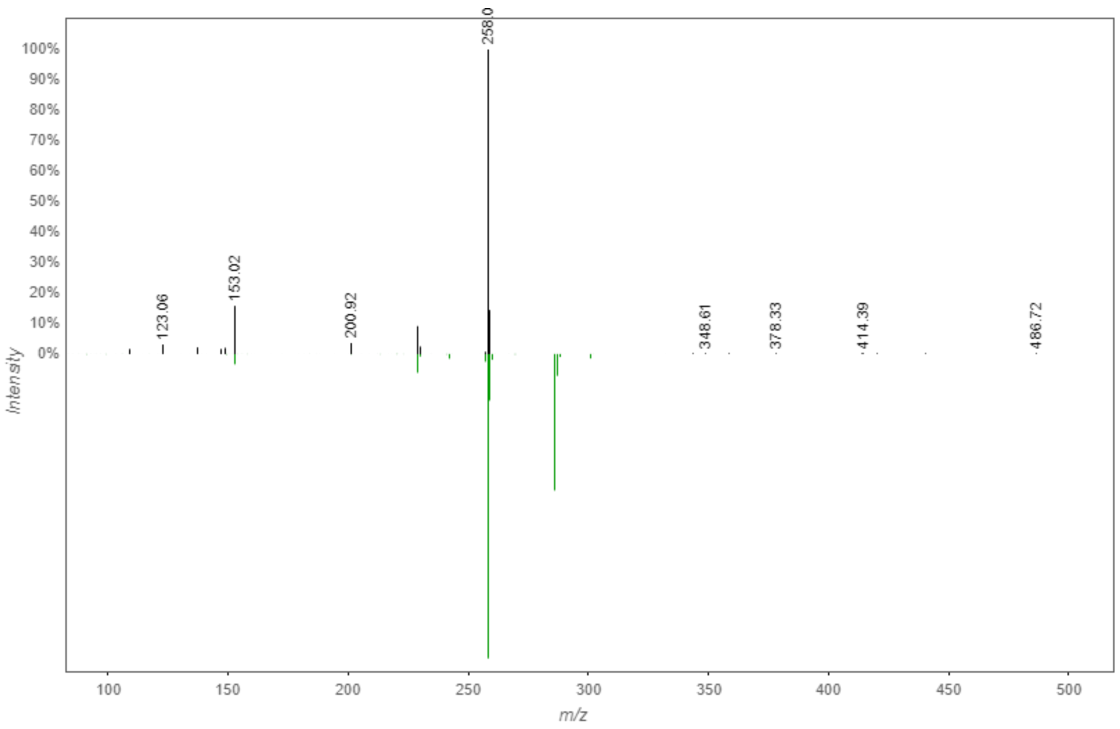

Fig S15. MS/MS match between GNPS database (green) and *Euterpe oleracea* Mart extract (black) for **8**.

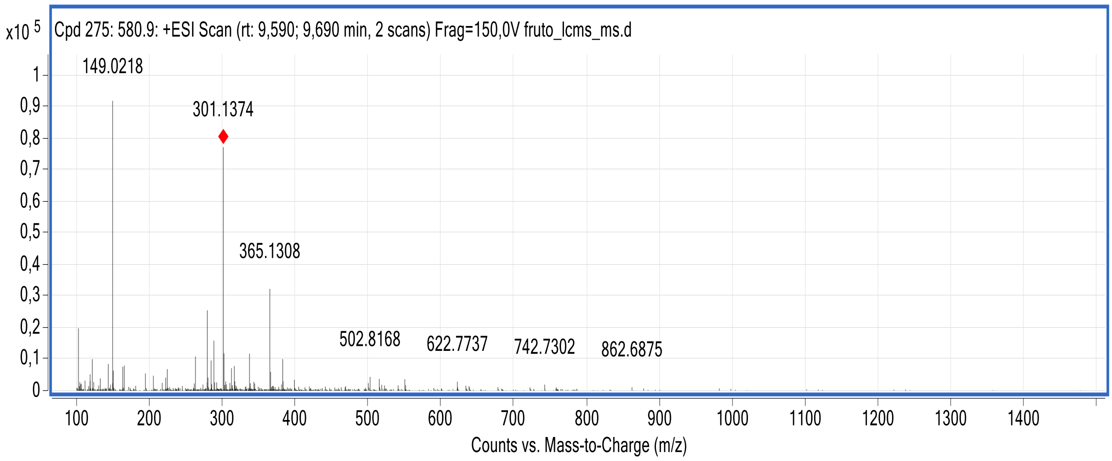

Fig. S16. Mass Spectrum of [M+H]$^{+}$ of **8**

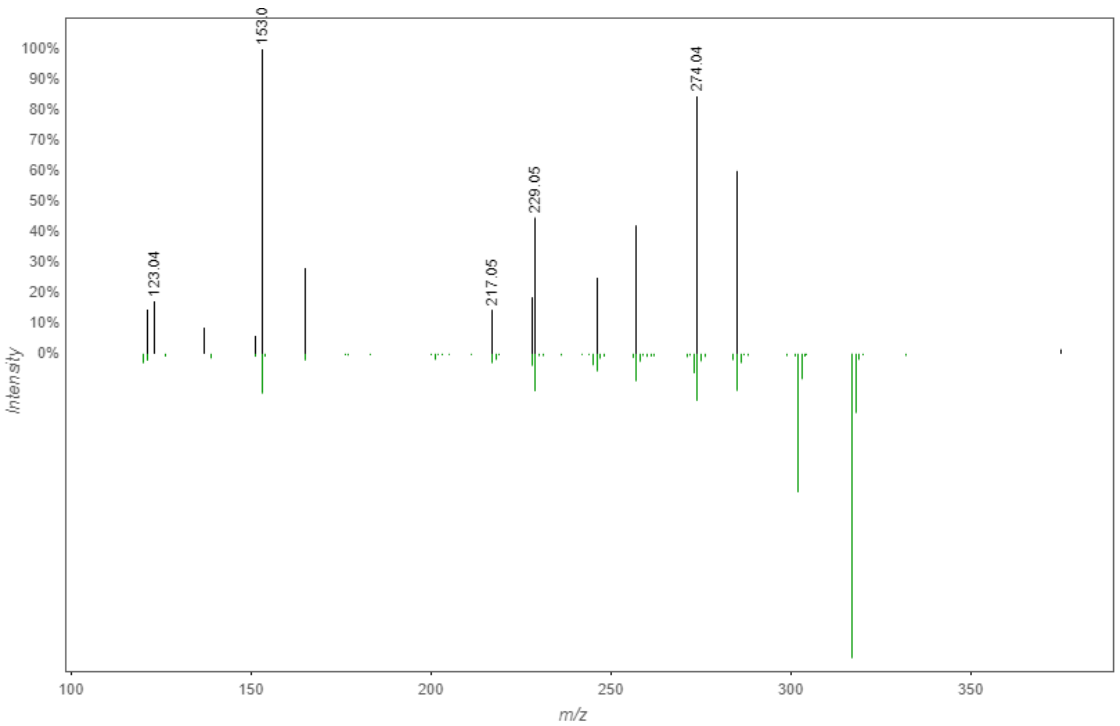

Fig S17 MS/MS match between GNPS database (green) and *Euterpe oleracea* Mart extract (black) for **9**.

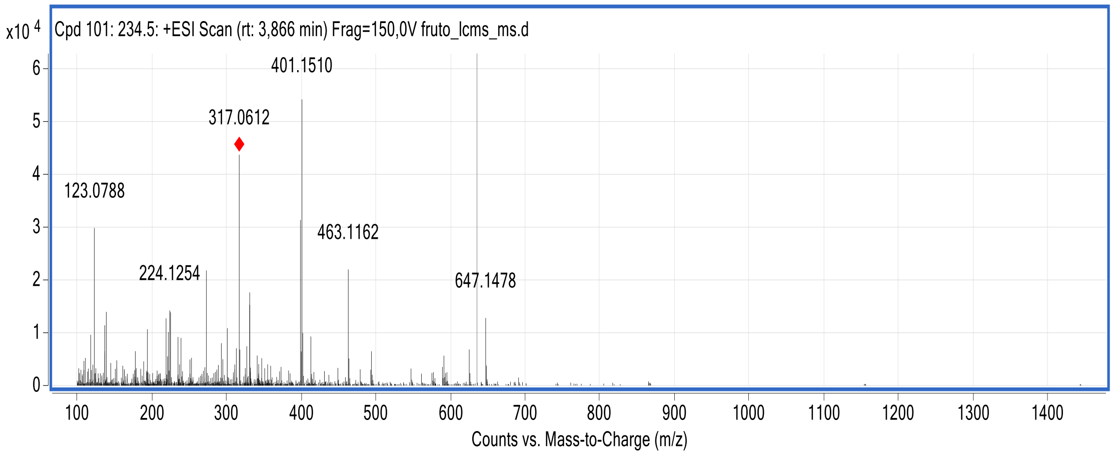

Fig. S18. Mass Spectrum of $[M+H]^+$ of **9**

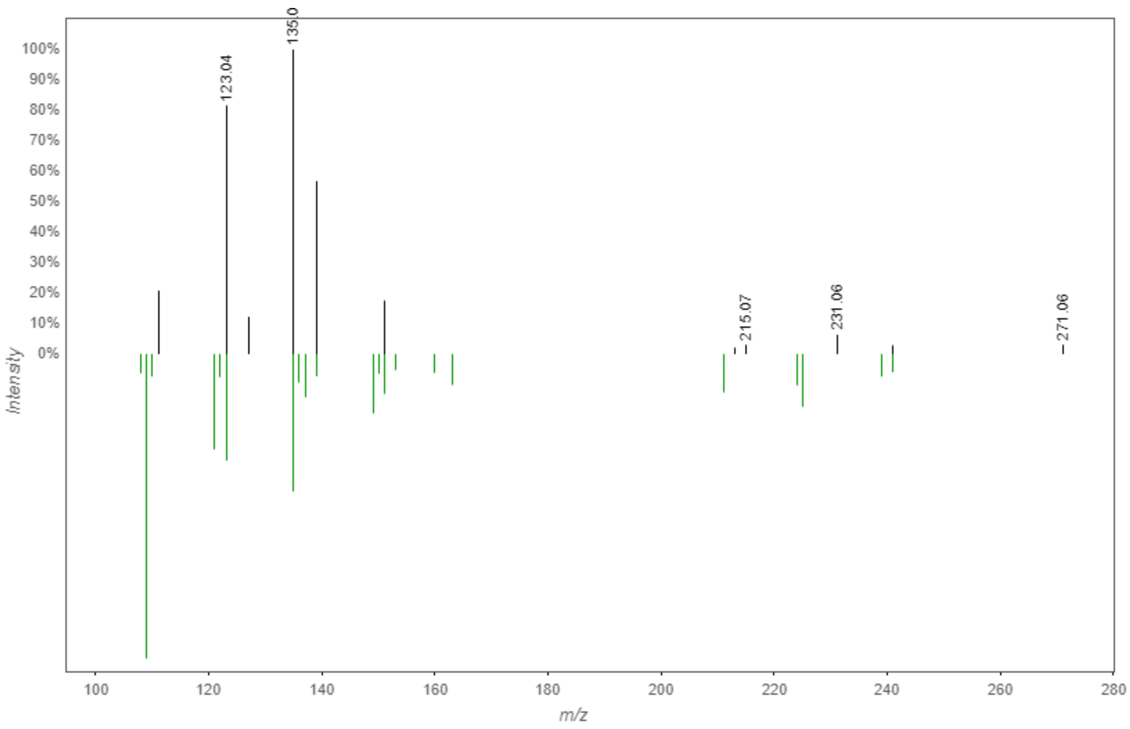

Fig S19. MS/MS match between GNPS database (green) and *Euterpe oleracea* Mart extract (black) for **10.**

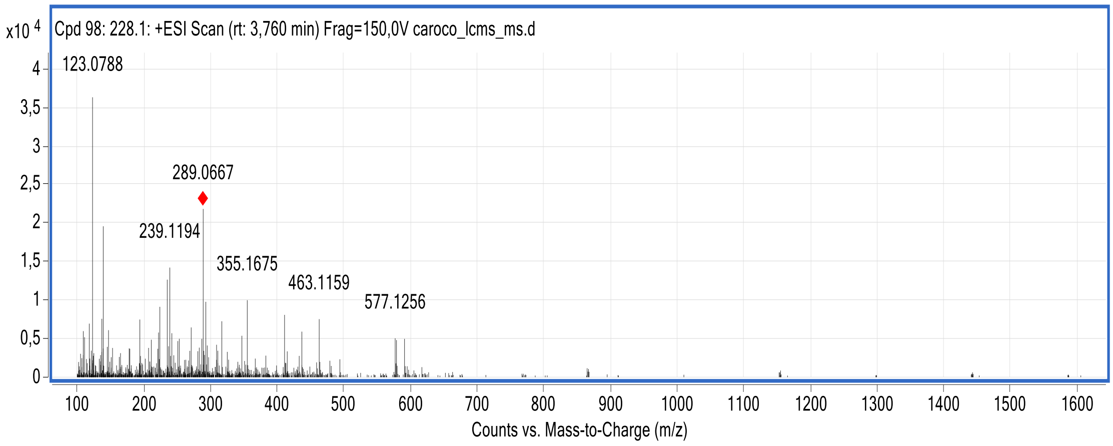

Fig. S20. Mass Spectrum of $[M+H]^+$ of **10**

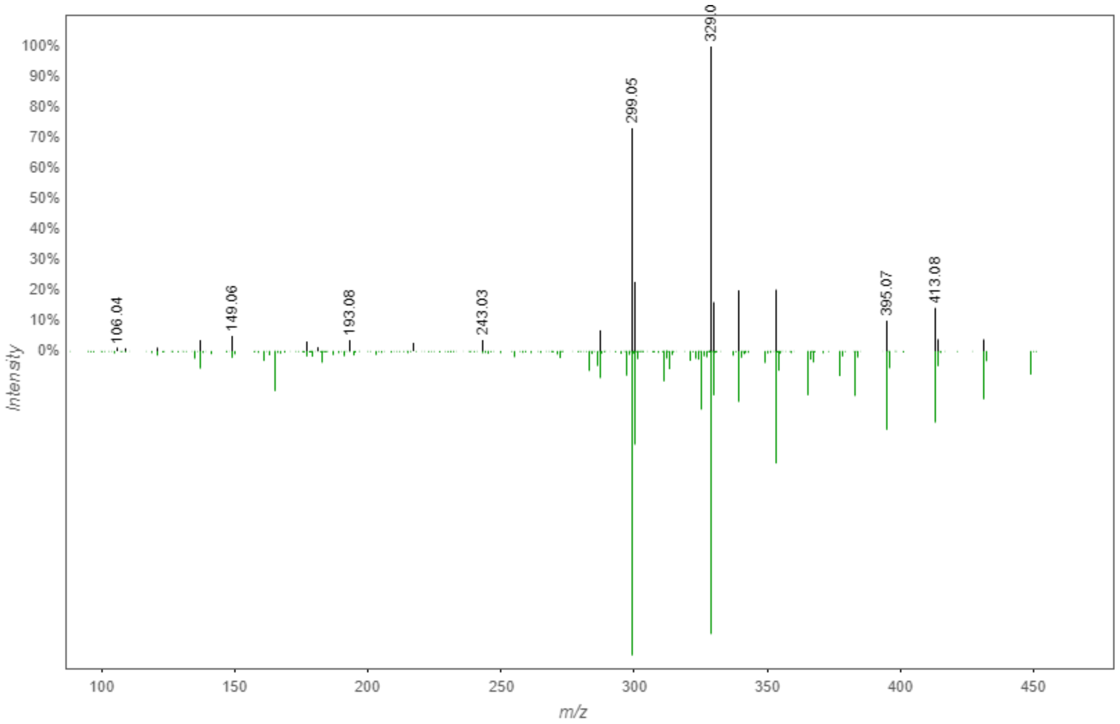

Fig S21. MS/MS match between GNPS database (green) and *Euterpe oleracea* Mart extract (black) for **11**.

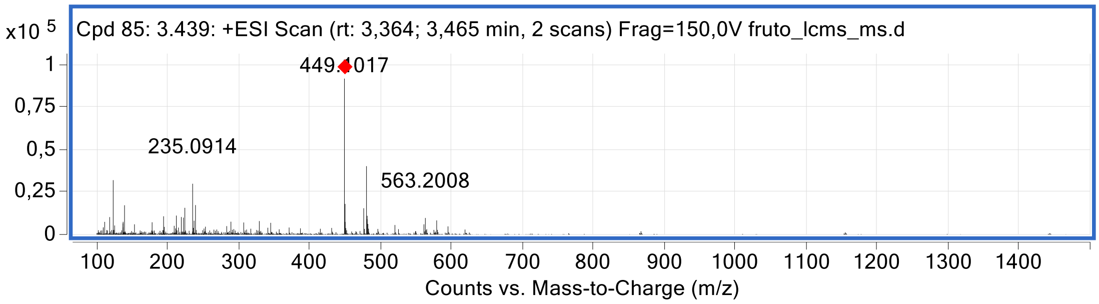

Fig. S22. Mass Spectrum of $[M+H]^+$ of **11**

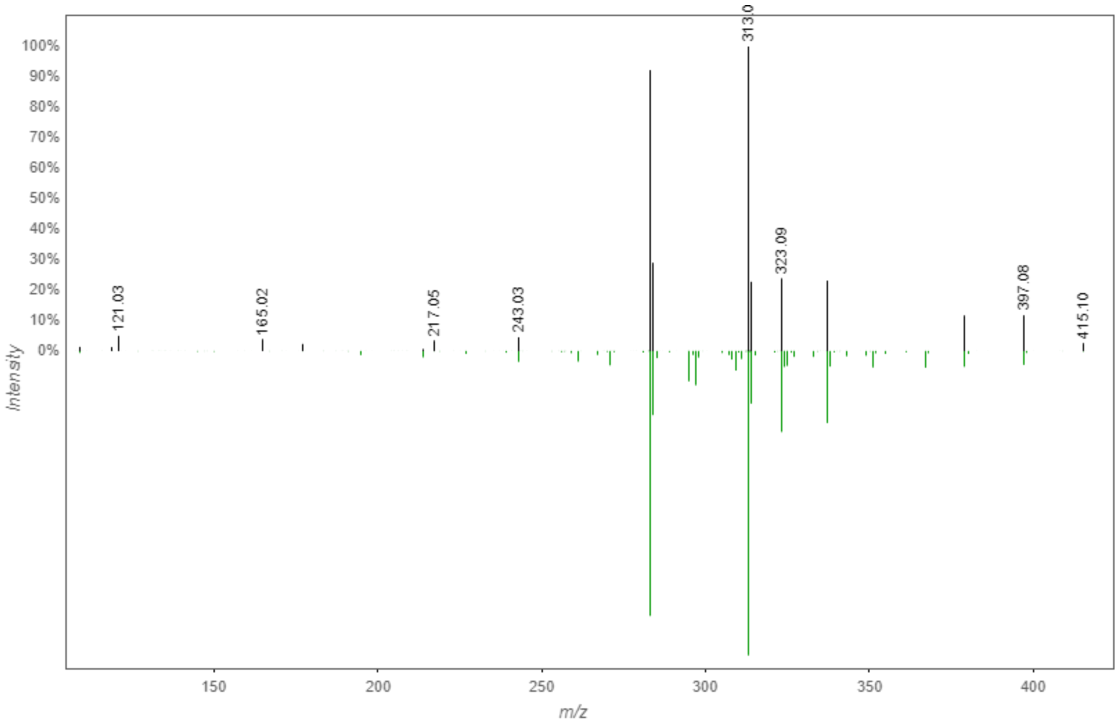

Fig S23. MS/MS match between GNPS database (green) and *Euterpe oleracea* Mart extract (black) for **12**.

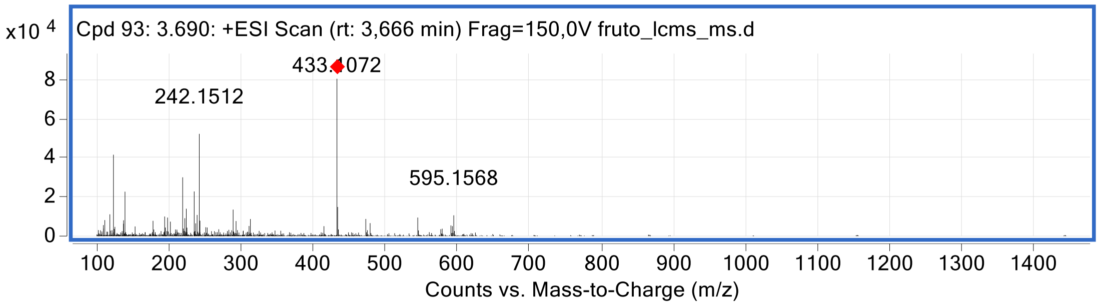

Fig. S24. Mass Spectrum of $[M+H]^+$ of **12**

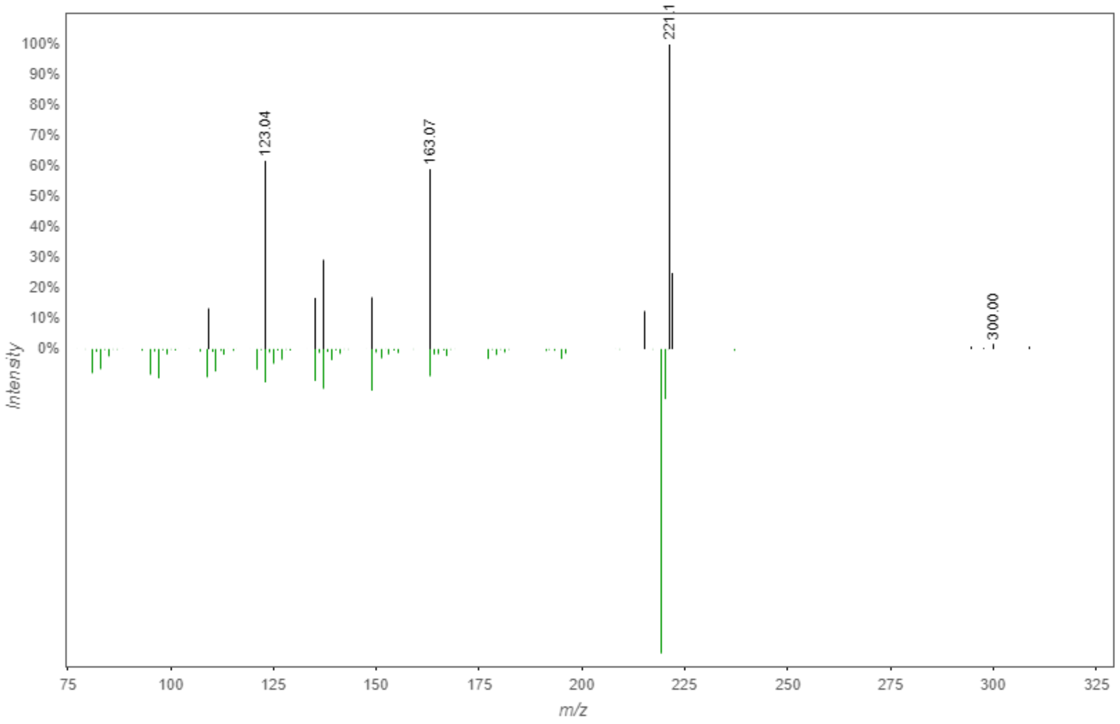

Fig S25. MS/MS match between GNPS database (green) and *Euterpe oleracea* Mart extract (black) for **13**

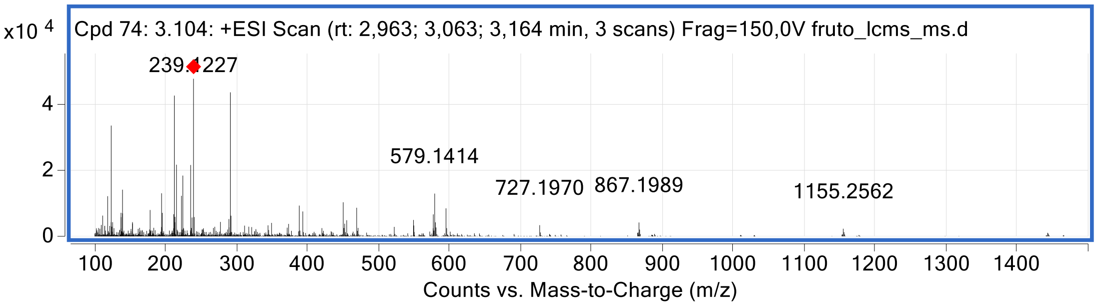

Fig. S26. Mass Spectrum of $[M+H]^+$ of **13**

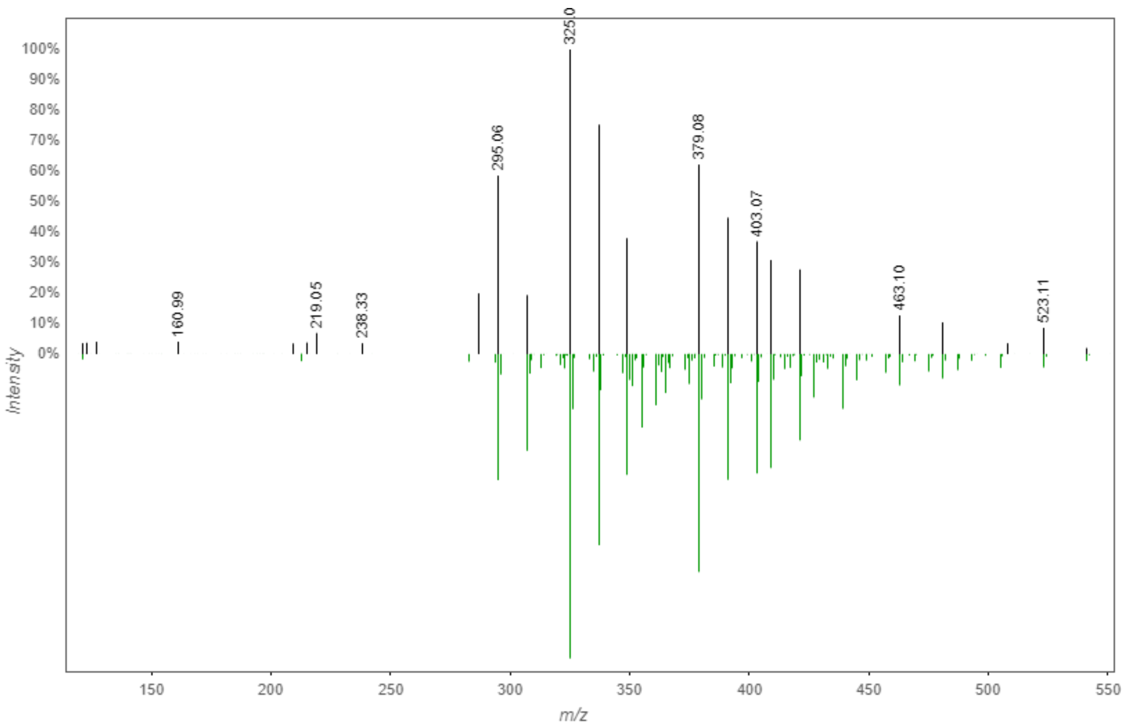

Fig S27. MS/MS match between GNPS database (green) and *Euterpe oleracea* Mart extract (black) for **14**

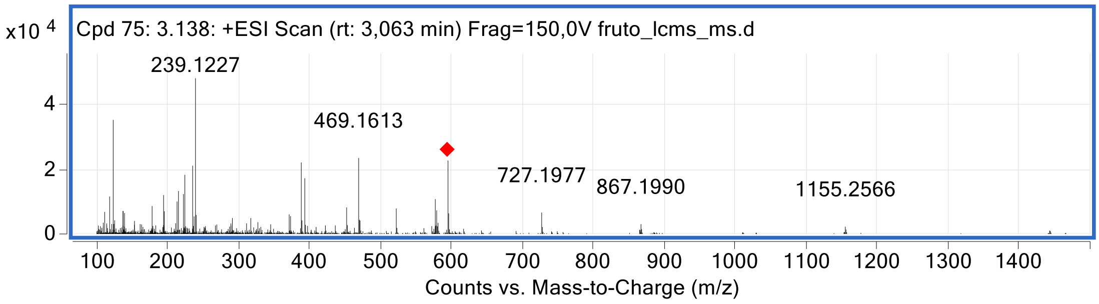

Fig. S28. Mass Spectrum of $[M+H]^+$ of **14**
